# High‐Temperature and High‐Electron Mobility Metal‐Oxide‐Semiconductor Field‐Effect Transistors Based on N‐Type Diamond

**DOI:** 10.1002/advs.202306013

**Published:** 2024-01-19

**Authors:** Meiyong Liao, Huanying Sun, Satoshi Koizumi

**Affiliations:** ^1^ Research Center for Electronic and Optical Materials National Institute for Materials Science (NIMS) 1‐1 Namiki Tsukuba Ibaraki 3050044 Japan; ^2^ Beijing Academy of Quantum Information Sciences No. 10 East Xibeiwang Road, Haidian Beijing 100193 China

**Keywords:** MOSFET, n‐type conductivity, semiconductor diamond

## Abstract

Diamond holds the highest figure‐of‐merits among all the known semiconductors for next‐generation electronic devices far beyond the performance of conventional semiconductor silicon. To realize diamond integrated circuits, both n‐ and p‐channel conductivity are required for the development of diamond complementary metal‐oxide‐semiconductor (CMOS) devices, as those established for semiconductor silicon. However, diamond CMOS has never been achieved due to the challenge in n‐type channel MOS field‐effect transistors (MOSFETs). Here, electronic‐grade phosphorus‐doped n‐type diamond epilayer with an atomically flat surface based on step‐flow nucleation mode is fabricated. Consequently, n‐channel diamond MOSFETs are demonstrated. The n‐type diamond MOSFETs exhibit a high field‐effect mobility around 150 cm^2^ V^−1^ s^−1^ at 573 K, which is the highest among all the n‐channel MOSFETs based on wide‐bandgap semiconductors. This work enables the development of energy‐efficient and high‐reliability CMOS integrated circuits for high‐power electronics, integrated spintronics, and extreme sensors under harsh environments.

## Graphical abstract

N‐channel diamond MOSFETs based on electronic grade phosphorous‐doped diamond are demonstrated. The MOSFET has the highest field‐effect electron mobility among all the wide‐bandgap semiconductors at high temperatures. This work enables the development of energy‐efficient and high‐reliability CMOS integrated circuits for high‐power electronics, integrated spintronics, and extreme sensors under harsh environments.

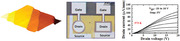

## 1 Introduction

Modern electronics is prevailed by silicon complementary metal‐oxide‐semiconductor (CMOS) technology. Nevertheless, silicon CMOS has been facing bottlenecks in the condition of high‐power density, high frequency, high temperature, and high radiation. Diamond is regarded as the ultimate semiconductor because of its superior characteristics compared to other semiconductors.^[^ ^1^ ^]^ Diamond CMOS devices have long been pursued to achieve performances beyond the capability of conventional silicon electronics. By using diamond electronics, not only the thermal management demands for conventional semiconductors be alleviated but also these devices are more energy efficient and can endure much higher breakdown voltages and harsh environments. On the other hand, with the development of diamond growth technologies,^[^ ^2^ ^]^ power electronics,^[^ ^3^ ^]^ spintronics,^[^ ^4^ ^]^ and microelectromechanical system (MEMS) sensors^[^ ^5^ ^]^ operatable under high‐temperature and strong‐radiation conditions, the demand for peripheral circuitry based on diamond CMOS devices has increased for monolithic integration.^[^ ^6^ ^]^ P‐type diamonds are readily accessible through bulk boron doping or surface transfer doping of a hydrogen‐terminated diamond surface.^[^ ^7^ ^]^ Nevertheless, in order to realize diamond CMOS, symmetrical doping control has to be achieved, as those accomplished for semiconductor silicon. Therefore, the development of diamond n‐MOS is in demand.

However, n‐channel diamond MOSFETs have long been an obstacle and have not been achieved yet due to the significant challenge in the growth of electronic grade high‐quality n‐type diamond. Until now, phosphorus has been recognized as the only reliable shallowest n‐type dopant at room temperature, despite the large covalent radius of P (1.08 Å) compared to that of C (0.77 Å) and high equilibrium formation energy (4–5.7 eV).^[^ ^8^ ^]^ However, due to the large carrier compensation ratio in phosphorus‐doped diamond, it has been difficult to achieve n‐type conductivity for low donor concentration of ≈10^17^ cm^−3^, hindering the development of n‐channel MOSFET. In addition to the defects induced by the larger radius of phosphorous than carbon, the incorporation of a large amount of hydrogen into the diamond epilayer during chemical vapor deposition (CVD) also passivates the phosphorous atoms and reduces the electrical conductivity.

In this study, electronic grade n‐type diamond with an atomically flat terrace was achieved based on the step‐flow lateral growth mode. N‐type diamond with low donor concentration of ≈10^17^ cm^−3^ is thus achieved without observing hopping conductivity. Consequently, n‐type diamond MOSFETs that can operate at 573 K have been successfully developed. The experimental field‐effect electron mobility at 573 K was around 150 cm^2^ V^−1^ s^−1^, the highest among all the wide‐bandgap semiconductors at high temperatures.

## 2 Results and Discussion

### 2.1 High‐Quality Phosphorus‐Doped Diamond Epilayer

**Figure 1:**
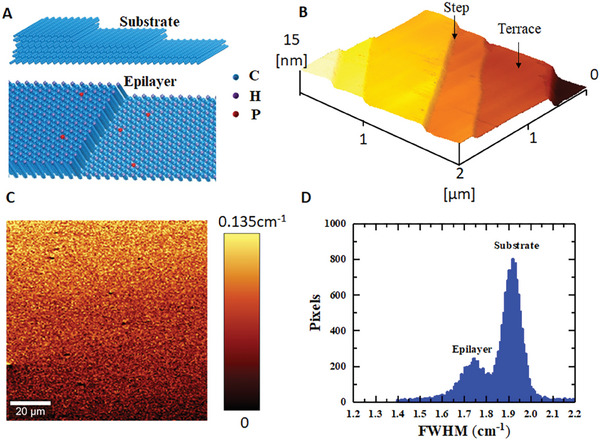
High‐quality lightly phosphorus‐doped n‐type diamond epilayer. A) Schematic of the atomic steps on the miscut diamond (111) substate (top) and the grown phosphorous‐doped epilayer with hydrogen‐termination (bottom). B) Surface morphology measured using AFM, showing an atomically smooth terrace. The steps are caused by the off angle of the diamond substrate. C) Two‐dimensional Raman mapping across the surface of the n^−^ diamond epilayer. The variation in the diamond feature peak centered at 1332.5 cm^−1^ is within 0.135 cm^−1^, demonstrating little stress in the diamond epilayer. D) Raman mapping in depth from the epilayer to diamond substrate. The full‐width at the half maximum of the epilayer is 1.75 cm^−1^, as low as those of high crystal quality intrinsic single‐crystal diamond layers.

We grew a phosphorus‐doped diamond epilayer via microwave plasma chemical vapor deposition (MPCVD) on a type‐Ib (111) oriented high‐pressure high‐temperature (HPHT) diamond substrate. The n‐type diamond contains two phosphorus‐doped epilayers: a lightly phosphorus‐doped n^−^ diamond epilayer for the device channel and a heavily phosphorus‐doped diamond epilayer for the Ohmic contact. The 600‐nm‐thick lightly doped n^−^ ‐layer diamond epilayer was grown directly on the HPHT diamond substrate. Following that, a 100‐nm‐thick heavily phosphorus‐doped *n^+^* – layer was deposited on the n^−^‐layer using a homemade MPCVD reactor, which enhanced the incorporation efficiency of phosphorus into the diamond epilayer. As grown diamond (111) has a unreconstructed monohydride‐terminated surface.^[^ ^9^ ^]^ The homoepitaxial growth of the n^−^‐type diamond on the diamond (111) substrate follows the step‐flow growth mode. Atomically flat terraces are formed (**Figure** **1**A), as observed by atomic force microscopy (AFM), shown in Figure 1B; Figure S1 (Supporting Information) with an average roughness (Ra) ≈0.1 nm. The average roughness of the terrace is ˂ 1 nm for a larger area of 10 × 10 µm^2^ (Figure S2, Supporting Information), despite the formation of steps in the entire epilayer. The terrace width is hundreds of nanometers, and the step height is ≈3 nm (Figure S3, Supporting Information). The surface steps are caused by the miscut of the HPHT diamond (111) substrate. The step‐flow growth mode resulted in a high‐quality n^−^ diamond epilayer. Raman mapping reveals that the feature peak of diamond exhibits little dispersion within 0.135 cm^−1^ and the full‐width at the half maximum (FWHM) of the diamond peak of the n^−^ diamond epilayer is centered at 1.75 cm^−1^, better than that of the HPHT diamond substrate of 1.95 cm^−1^ (Figure 1C,D). The stress in the n^−^ layer is as low as ‐12 MPa^[^ ^10^ ^]^ and the crystal quality is comparable to those of homoepitaxial diamond layers grown on (100) diamond substrates.^[^ ^11^ ^]^ The lateral distribution of the phosphorus concentration in the CVD diamond epilayer was uniform if the compressive stress is assumed to be caused primarily by the incorporation of phosphorus atoms. The phosphorus concentrations of n^+^/n^−^ diamond on the diamond substrate were measured using secondary ion mass spectrometry (SIMS), as shown in Figure S4 (Supporting Information). The 100‐nm‐thick *n^+^* layer has a phosphorus concentration of ≈10^20^ cm^−3^. The 600‐nm‐thick lightly doped n^−^ ‐layer diamond epilayer has a phosphorus concentration of *N*_D_ ≈10^17^ cm^−3^. A uniform distribution of phosphorus concentration along the growth direction could be observed in the SIMS data. Additionally, the SIMS depth profile shows that the hydrogen content is controlled at a noise level of 10^17^ cm^−3^. The well‐controlled incorporation of phosphorus and hydrogen atoms into the diamond epilayer implies a high crystal quality of the diamond epilayer, which is essential for achieving n‐type conductivity. In addition, no nitrogen‐vacancy related luminescence was detected from the epilayer.

The electron concentration strongly depends on the temperature because of the deep nature of phosphorus in diamond. The electron concentration is calculated as follows:

(1) $$\frac{n\left(n+N_{A}\right)}{N_{D}-N_{A}-n}=\frac{N_{C}}{g}\;\exp\left(-\frac{E_{D}}{k_{B}T}\right)$$

where *n* denotes the free electron concentration in the conduction band, *N*_D_ denotes the phosphorous concentration, *N*_A_ indicates the compensating acceptor density, *N*_C_ refers to the effective conduction band density of states, *g* indicates the degeneration factor of the donors, *E*_D_ denotes the activation energy of the donors, *k*_B_ is the Boltzmann constant, and *T* indicates the temperature. The electron density is ≈10^10^ cm^−3^ at 300 K and increased by four orders of magnitude at 573 K for *N*_D_ ≈10^17^ cm^−3^ (Figure S5, Supporting Information). The compensating acceptor concentration *N*_A_ is ≈2 × 10^16^ cm^−3^. At room temperature, the electron mobility measured by the Hall effect is ≈623 cm^2^ V^−1^ s^−1^.^[^ ^12^ ^]^ The lightly doped n^−^‐layer exhibits a high electron mobility of 212 cm^2^ V^−1^ s^−1^, even at 573 K (Figure S6, Supporting Information). The resistivity of the lightly doped n^−^ ‐layer film is ≈10^6^ Ω cm at room temperature and decreased to 100 Ω cm at 573 K (Figure S7, Supporting Information). The thermal activation energy, *E*_D_ is ≈0.57 eV.

### 2.2 Electrical Properties of N‐Type Diamond MOSFETs

**Figure 2:**
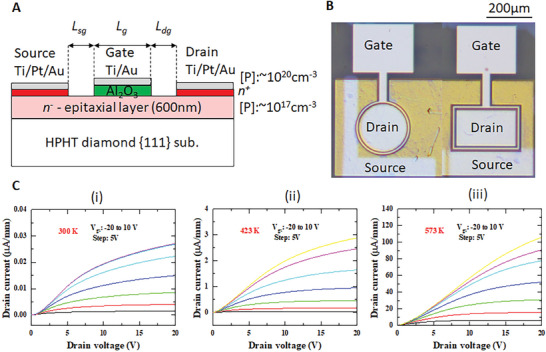
MOSFETs based on phosphorous doped n‐type diamond and the electrical characteristics with temperatures up to 573 K. A) Schematic of the MOSFETs. The n^+^ diamond layer is used to reduce the source and drain contact resistance. The n^−^ diamond layer serves as the channel. B) Optical image of the diamond MOSFETs. C) Transistor properties at 300, 423, and 573 K. The drain current increases by nearly four orders of magnitude from room temperature to 573 K.

We fabricated n‐channel diamond MOSFETs with two types of geometries: rectangular and Corbino (**Figure** **2**; Table S1, Supporting Information). The source (S) and drain (D) contacts were formed on the heavily phosphorus‐doped n^+^ layer, which was annealed Ti (50 nm)/Pt(10 nm)/Au(60 nm). The electrical resistivity of the heavily doped n^+^ ‐diamond is ≈80 Ω cm at RT and 20 Ω cm at 573 K.^[^ ^13^ ^]^ A lightly phosphorus‐doped n^−^‐layer was used as the channel of the MOSFETs. The top heavily doped n^+^ diamond layer between the S and D electrodes was etched in oxygen plasma until it reached the lightly doped layer. The gate oxide was 30‐nm‐thick Al_2_O_3_ deposited via atomic layer deposition (ALD) at 473 K. The gate metal consisted of a 10‐nm‐thick Ti layer covered by a 60‐nm‐thick Au layer. The gate lengths (*L*_g_) are 5 and 10 µm, and the source‐drain (*L*_sg_) and drain‐gate space (*L*_dg_) are 5 and 10 µm, respectively. The inner and outer diameters of the gate for the Corbino MOSFETs are 220 and 230 µm, respectively. For the rectangular MOSFET (device No. 1) studied here, *L_g_* is 5 µm, *L*_sg_ = *L*_dg_ = 10 µm, and the gate width is ≈900 µm. Figure 2A,B shows schematic and optical images of the n‐type diamond MOSFETs, respectively. The electrical characterization of the MOSFETs was performed in a vacuum chamber (10^−3^ Pa) using a semiconductor parameter analyzer and a shielded probe station. The temperature of the MOSFETs was increased from room temperature to 573 K for electrical characterization.

The dependence of the drain current (*I*_d_) normalized by the gate width on the drain voltage (*V*_ds_) of the rectangular MOSFET is shown in Figure 2C. Here, we show the *I*_d_−*V*_ds_ characteristics measured at three temperatures: 300 K (RT), 423 K (150 °C), and 573 K (300 °C). The gate voltage (*V*_gs_) of the MOSFET was varied from −20 to 10 V in steps of 5 V. The drain current was well modulated by the gate voltage, displaying a typical transistor action with an n‐type channel. The maximum drain current (*I*_d,sat_) at *V*_ds_ = 20 and *V*_gs_ = 5 V is ≈0.027 µA mm^−1^ at 300 K. However, further increasing *V*_gs_ > 5 V resulted in little improvement in the drain current owing to the high series resistance. Temperature‐dependent *I*_d_−*V*_ds_ characteristics were obtained until the drain current of the MOSFET stabilized over time at a certain temperature. As illustrated in Figure 2C(ii),(iii), the drain current markedly increases with the temperature, owing to the thermal ionization of phosphorus. At high temperatures and *V*_ds_ = 20 V and *V*_gs_ = 10 V, the drain current increases to 2.9 µA mm^−1^ at 423 K and 105 µA mm^−1^ at 573 K, which is two and four orders of magnitude higher than that at 300 K, respectively. This is consistent with the dependence of electrical resistivity on temperature (Figure S7, Supporting Information). The drain voltage required to reach the saturation increases with temperatures and gate voltage, that is *V*_ds_ is >30 V for the saturation at 573 K and *V*_gs_ = 10 V. The on‐resistance is estimated to be ≈5 GΩ mm at RT, which reduced to 160 kΩ mm at 573 K at *V*_gs_ = 10 V. The variations in the electrical characteristics of the MOSFET at other temperatures are shown in Figures S8–S12 (Supporting Information). The dependence of the drain current on the measured temperature is presented at different gate voltages (Figure S13, Supporting Information). The drain current increases exponentially with the temperature. A fitting using the Arrhenius equation of the temperature‐dependent drain current provides a thermal activation energy of 0.45 eV.

**Figure 3:**
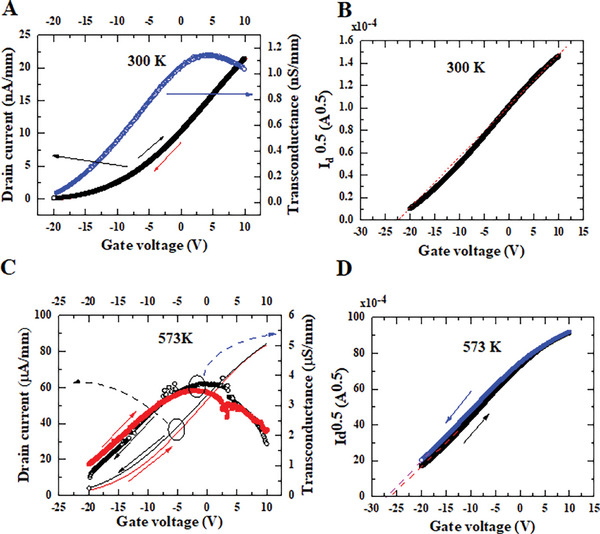
Transfer properties of the n‐channel diamond MOSFET. A) I_d_ versus V_gs_ characteristics and transconductance at V_ds_ = 15 V and at 300 K. No hysteresis is observed. B) I_d_^0.5^ versus V_gs_ curve at 300 K. The plot is not a straight line, revealing the series resistance effect. C) I_d_ versus V_gs_ characteristics and transconductance at V_ds_ = 15 V and at 573 K. Little hysteresis is observed, while the threshold voltage changes slightly. D) I_d_^0.5^ versus V_gs_ curve at 573 K. A nonlinear behavior is observed, revealing that the ideal MOSFET model does not work well.

**Figure 4:**
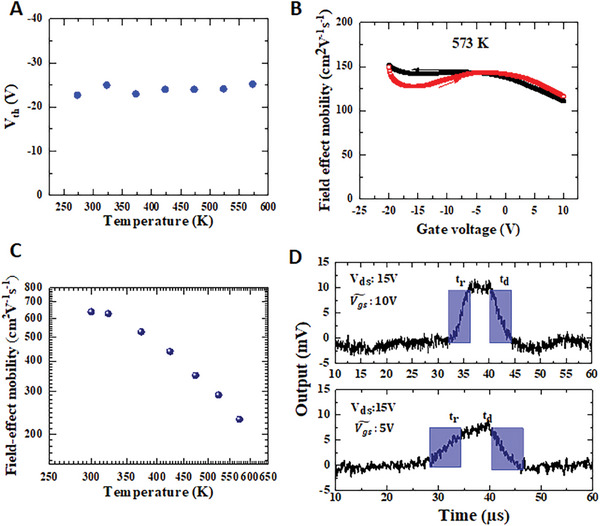
Experimental and theoretical field‐effect electron mobility. A) Threshold voltages (V_th_) extracted by the graphic method based on the transfer curves. V_th_∼‐ 25 V changes little with temperature. B) Experimental field‐effect mobility as V_gs_ at 573 K by the saturation model, which is slightly gate‐dependent. C) Theoretical field‐effect mobility considering the thermal ionization effect of the donor and series resistance. The mobility decreases as the temperature increases and is comparable to those measured by Hall effect. D) Switching speed at 573 K of the n‐type MOSFET. The rising and decay times are < 5 µs at V_ds_ = 15 V when the radio‐frequency signal amplitude at the gate is 10 V. The switching speed is slower for a smaller gate amplitude, when the radio‐frequency signal amplitude applied to the gate is 5 V.

The transfer characteristics of the MOSFET or the gate voltage‐dependent drain current are shown in **Figure** **3**A at 300 K and Figure 3C at 573 K at *V*_ds_ = 20 V in the saturation region. The ratio of the drain current at gate voltages of 10 and −20 V is >  200 at RT and 100 times at 573 K at *V*_ds_ = 20 V. Similar to MOSFETs based on boron‐doped diamond, the n‐type diamond MOSFET exhibits a deep depletion mode.^[^ ^14^ ^]^ Little hysteresis is observed in the transfer curves at temperatures below 473 K. Only slight hysteresis is observed at 573 K. The maximum transconductance g_m_ is around 0.012 µS mm^−1^ at 300 K and around 4 µS mm^−1^ at 573 K. The threshold voltages (*V*_th_) were extracted using the graphic method of *V_gs_* versus *I*_d_^0.5^ (Figure 3B,D), which is ≈−25 V. There is little change in *V*_th_ with the gate‐sweeping direction or temperature (**Figure** **4**A). Other devices with different geometries were also measured, and their electrical properties are shown in Figures S14–S17 (Supporting Information) and similar n‐channel behavior was observed. The electrical performances, such as the maximum saturated drain current, maximum transconductance, threshold voltage, and temperature‐dependent behavior, are comparable with those of MOSFETs with similar dimensions (Table S1, Supporting Information).

### 2.3 Modeling the Field‐Effect Electron Mobility

For an ideal MOSFET in which the mobility does not depend on the gate voltage or series resistance, the effective electron mobility µ_eff_ could be calculated using the quadratic model in the saturation region as follows:

(2) $$I_{d,\;sat}=\frac{W_{g}}{2L_{g}}\;C_{ox}\mu_{eff}{\left(V_{gs}-V_{th}\right)}^{2}$$

where I_d,stat_ denotes the drain current in the saturation region and C_ox_ denotes the capacitance of the gate oxide. We attempted to determine the field‐effect electron mobility using Equation (2). However, the field‐effect mobility is as low as 0.02 cm^2^ V^−1^ s^−1^ at 300 K, significantly (almost 3000 times lower) deviated from the reasonable value measured by Hall measurements. Substituting the series resistance into Equation (2) does not cause an essential change in mobility.^[^ ^1a^ ^]^ An increase in the field‐effect electron mobility is observed as the temperature increases, which is unreasonable. At 573 K, the electron mobility calculated using Equation (2) is ≈150 cm^2^ V^−1^ s^−1^ (Figure 4B), which is much higher than those of n‐channel MOSFETs based on SiC, GaN, and Ga_2_O_3_^[^ ^15^ ^]^ at high temperatures. Note that the mobility is underestimated even at 573 K owing to the large series resistances in the source/drain and drift regions and the partial thermal ionization of phosphorus in diamond.

To precisely evaluate the field‐effect mobility, we consider the i) thermal ionization efficiency of the phosphorus donor, ii) series resistance, and iii) mobility degradation factors (i.e., defects scattering). Therefore, in the linear region, the drain current (I_d_) can be expressed as^[^ ^16^ ^]^

(3) $$I_{d}=\frac{\gamma W_{g}}{L_{g}\left(1+M\right)}\;\left[V_{gs}-V_{th}-\alpha \frac{V_{d}}{2}\right]V_{ds}$$

where M denotes the donor occupancy factor, a ratio of bound donor charge to channel charge that reflects the ionization rate of the donor and is related to the quasi Femi level of electrons. Here, we assume that M is independent of the channel depth. Note that M has the analytical form different from the ratio of free electron density to doping density (Supporting Information). The larger M is, the smaller the ratio of the free electron density to doping density is. α is the factor lowering the drain current related to the donor concentration, which is ≈1.1 here. γ includes the factors of θ and η that modulate the mobility (Supporting Information). The factor θ is related to conventional carriers scattering and the effect of series resistance. The effect of the drain voltage on the carrier mobility is connected to the parameter η included in γ. The none zero η is mostly due to the irregularity in the nanoscale/microscale mesa structure due to oxygen etching. The electron mobility was simulated in a region well above the threshold voltage. The saturation of the carrier velocity is not considered because of the small drain voltage, large distance between the source and drain, and large gate length. The thermal ionization of phosphorous in diamond was set at 0.57 eV for the simulation. We obtained the field‐effect mobility of the n‐type MOSFET at a gate voltage of 0 V, as shown in Figure 4C. The mobility was simulated to be ≈638 cm^2^ V^−1^ s^−1^ at 300 K, which decreased to ≈200 cm^2^ V^−1^ s^−1^ at 573 K by considering the donor occupancy factor M and series resistance. For an ideal MOSFET, M decreases with increasing current and ultimately reaches zero. M was calculated as 2278 at 300 K, which decreases to ≈4 at 573 K (Figure S18, Supporting Information), revealing the n‐type diamond MOSFET depletion mode. It should be noted that the compensating acceptor effect was not considered. The simulated characteristics of the drain voltage versus drain current are displayed in the SM (Figure S19, Supporting Information) by considering the donor occupancy factor and series resistance. A slight discrepancy exists in the low drain voltage region, mostly owing to the barrier between the n^+^ and n^−^‐layers in the S and D electrodes. We note that the simulation was conducted by assuming the entire n^−^ – layer conductive. Considering the Femi‐level pining of oxygen‐terminated phosphorous‐doped n‐type (111) diamond^[^ ^17^ ^]^, subsurface depletion of the channel occurs. The simulation from a metal‐Schottky FET by using a similar n^−^‐channel diamond layer revealed that the sub‐depletion layer was ≈50 nm. For the n‐type MOSFET, the Femi‐level pining along the fixed charges in the insulator modifies the Femi potential in the simulation. Detailed experimental and theoretical investigation should be conducted to disclose the effect of defects states in the future.

Currently, p‐channel diamond MOSFETs have been extensively developed and a routine fabrication process has been established. Owing to the lack of diamond n‐MOSs, a complementary circuit has been reported to be accomplished using diamond p‐MOSs and III‐nitride n‐MOSs.^[^ ^18^ ^]^ Although this is a promising strategy, all‐diamond CMOS is the ultimate pursuit to fully exploit the figure‐of‐merit of diamond, particularly for electronics that operate under harsh environments (high temperatures and strong radiation). For high‐frequency operation, compared with H‐terminated transistors with a cutoff frequency of over GHz,^[^ ^1d^ ^]^ the series resistance is still large for n‐type diamond MOSFETs, which is over 10^9^ Ω mm^−1^ at room temperature. Thus, the operating speed was limited to the kilohertz range. Nevertheless, at temperatures > 573 K, the series resistance decreases by over three orders of magnitude. The switching speed is ˂ 5 µs (Figure 4D), which could also be tuned by the signal applied to the gate. The switching speed is faster for a larger gate amplitude due to the increase in the channel conductivity. By optimizing the device geometries, such as the reduction of the drift region space and gate length, the operation frequency can exceed the megahertz range, comfortably satisfying the requirements of mixed‐signal circuits for radiation detectors and MEMS sensors.^[^ ^5^, ^19^ ^]^ In addition, n‐type diamonds can stabilize the negatively charged nitrogen‐vacancy (NV^−^) state, greatly improving sensitivity. Thus, diamond CMOS‐integrated NV centers are favorable for the development of diamond spin electronics that require dedicated controllability and integrity to scale up the quantum sensing protocol.^[^ ^20^ ^]^ The deep nature of the phosphorus in diamond benefits the generation of surface p‐type conductivity in a lightly phosphorus‐doped diamond epilayer with hydrogen termination. Thus, a diamond CMOS based on a planar process on lightly doped n‐type diamond can be achieved. By using MEMS technology^[^ ^21^ ^]^ to engineer the band structure,^[^ ^22^ ^]^ the performance of n‐type diamond MOSFETs can be further improved. This study sheds light on monolithically integrated diamond chips, in which electronics, spintronics, and sensors are based on diamond.

## 3 Conclusion

In conclusion, n‐type channel diamond MOSFETs were demonstrated on phosphorus‐doped homoepitaxal (111) diamond epilayer. The n‐type (111) diamond epilayer was grown based on a step‐flow nucleation mode, enabling the precise control of the crystal quality and the donor distribution. The n‐MOSFET showed a high mobility ≈150 cm^2^ V^−1^ s^−1^ at 573 K, a significant feature over other wide‐bandgap semiconductors at high temperatures. The excellent high‐temperature performance offers the route to develop diamond CMOS circuits for high‐power electronics, integrated spintronics, and extreme sensors under harsh environments.

## 4 Experimental Section

### Growth of Phosphorous Doped N‐type Diamond

The phosphorus‐doped diamond epilayers were grown by using a microwave plasma‐assisted chemical vapor deposition (MPCVD) on a type‐Ib (111) high‐pressure high‐temperature (HPHT) diamond substrate with a 3° misorientation. The n‐type diamond contained two phosphorus‐doped epilayers: a lightly doped diamond epilayer for the device channel and a heavily doped diamond epilayer for the Ohmic contact. The lightly doped n^−^‐diamond epilayer was directly grown on the HPHT diamond substrate. The gas pressure, microwave power, and substrate temperature were 100 Torr, 500 W, and 920 °C, respectively. The heavily phosphorus‐doped n^+^‐diamond epilayer was grown by using a homemade MPCVD reactor to enhance the incorporation efficiency of phosphorus into diamond. The methane concentration was 0.05%. The phosphorus to carbon ratio was 10 000 ppm for the heavily phosphorus‐doped diamond epilayer. The growth duration was 15 min. The impurity levels of the lightly and heavily phosphorus‐doped diamond were ≈10^17^ and 10^20^ cm^−3^, respectively, which were measured by secondary ion mass spectroscopy (SIMS). The thickness of the n^−^ – layer and *n^+^* – layer was 600 and 100 nm, respectively.

### Raman Spectroscopy Imaging

Confocal Raman imaging was conducted on the n^−^ diamond epilayer by using a WITec α−300R spectroscopy. The wavelength and power of the excitation laser were 532 nm and 20 mW, respectively. The spectrometer was equipped with a 1800 Lmm^−1^ monochromator grating and a cooled charge coupled device detector. All measurements were taken at room temperature using a backscattering geometry. The crystal axis of diamond with z = [001], x = [010], and y = [100] directions is set to be the coordinate of X–Y–Z for the sample stage. The electric field of the incident beam is along the x = [010] direction of diamond. The wavenumber was first calibrated by using natural type‐IIa single‐crystal diamond before recording the Raman spectra of the n^−^ diamond epilayer. The peak position of the first‐order diamond line was set as 1332.5 cm^−1^ for the type‐IIa diamond. To achieve a high spatial resolution, the objective lens with a ×50 magnification was adopted. The numerical aperture of the lens was 0.75. According to the Rayleigh criterion, the lateral spatial resolution was 0.43 µm. The depth resolution was ≈2 µm. However, the actual resolution can be much smaller based on the statistical analysis. A thicker lightly phosphorous‐doped diamond epilayer grown by the same conditions was used for the Raman spectroscopy measuremnts.

### Fabrication of N‐Type Diamond MOSFETs

Two kinds of geometries: circular and rectangular shapes were adopted (Table S1, Supporting Information). The n‐type diamond epilayer was boiled in an acid mixture of HNO_3_ and H_2_SO_4_ to remove the surface contaminations and oxidize the diamond surface. The n‐type diamond MOSFETs were fabricated through a lithography process. First, source (S) and drain (D) contacts Ti (50 nm)/Pt(10 nm)/Au(60 nm) were formed on the n^+^ diamond layer by using an electron beam deposition and annealed at 773 K for 30 min in a high vacuum chamber. Second, the top heavily doped n^+^ diamond layer between the S and D electrodes was etched in an oxygen plasma until reaching the lightly doped one. The S and D electrodes were used as the mask for the reactive ion etching without an additional photolithography. The RIE conditions were: an oxygen flow rate of 90 sccm, a rf power of 800 W, and a bias of 20 W. The etching rate was ≈50 nm min^−1^. Then, a 30 nm‐thick Al_2_O_3_ was deposited by atomic layer deposition (ALD) at 473 K as the gate oxide on the patterned parts between the S and D electrodes. The gate metal was a 10 nm‐thick Ti layer covered by a 60 nm‐thick Au layer.

### Electrical Characterization

The current–voltage (*I–V*) characteristics of the n‐type diamond MESFET were measured by using a semiconductor parameter analyzer (Keithley 2602B) and a three‐probe station. The source is grounded, and no bias is applied to the back of the type‐Ib diamond substrate. The gate leakage was simultaneously recorded while measuring the MOSFETs properties. The measurements were conducted under a vacuum chamber. The temperature of the sample was elevated from room temperature to 573 K. The temperature‐dependent electrical properties were recorded until stable currents were obtained.

## Conflict of Interest

The authors declare no conflict of interest.

## Supporting information

Supporting Information

## Data Availability

The data that support the findings of this study are available from the corresponding author upon reasonable request.
