# Supplementary material for: High‐Temperature and High‐Electron Mobility Metal‐Oxide‐Semiconductor Field‐Effect Transistors Based on N‐Type Diamond
Source: Adv Sci (Weinh). 2024 Jan 19;11(13):2306013. doi: 10.1002/advs.202306013 (PMC10987156; doi:10.1002/advs.202306013)
Supplement: Supplementary file 1 — Supporting Information [file ADVS-11-2306013-s001.pdf]

ADVANCED SCIENCE Open Access

Supporting Information

for *Adv. Sci.*, DOI 10.1002/advs.202306013

High-Temperature and High-Electron Mobility Metal-Oxide-Semiconductor Field-Effect Transistors Based on N-Type Diamond

Meiyong Liao, Huanying Sun and Satoshi Koizumi**

Supporting Information

High-temperature and high-electron mobility metal-oxide-semiconductor field-effect transistors based on n-type diamond

Meiyong Liao[1], Huanying Sun[1,2], Satoshi Koizumi[1]*

Modeling the transistor properties

Different from the conventional semiconductors in which the dopants are fully ionized at room temperature, phosphorous in diamond forms a deep donor with a thermal activation energy of 0.57 eV. We include the effects of (i) temperature dependent thermal ionization of the phosphorous donor, (ii) series resistance, and (iii) mobility degradation factors (i.e. interface scattering). In the linear region with gate voltage V_{gs} larger than the threshold voltage V_{th}, the drain current (I_d) can be expressed as [1]

$$I_d = \frac{\gamma W_g}{L_g(1+M)}[V_{gs} - V_{th} - \alpha\frac{V_{ds}}{2}]V_{ds} \qquad S(1)$$

$$M = \frac{g_d N_d \exp\,[(E_C - E_D)/k_B T]}{N_C\left\{g_d \exp\left[-\frac{E_D - E_F}{k_B T}\right] + 1\right\}} \qquad S(2)$$

$$\gamma = \frac{\mu C_{ox}}{1+\theta\left(V_{gs} - V_{th} - \frac{V_{ds}}{2} + K\sqrt{2\emptyset_F}\right) + \eta V_{ds}} \qquad S(3)$$

$$\alpha = 1 + \frac{0.5K}{\sqrt{2\emptyset_F}}[1 - \frac{1}{1.41 + 0.43\times 2\emptyset_F}] \qquad S(4)$$

$$K = \frac{\sqrt{2qN_d\varepsilon_0\varepsilon_{dia}}}{C_{ox}} \qquad S(5)$$

$$\emptyset_F = \frac{k_B T}{q}\ln\left(\frac{N_d}{n_i}\right) \qquad S(6)$$

$$C_{ox} = \frac{\varepsilon_0\varepsilon_{ox}}{t_{ox}} \qquad S(7)$$

where M is the donor occupancy factor, reflecting the ionization rate of the donor. In detail, $M = Q_d/Q_n$, where Q_d is the charge per unit area of occupied donor sites and Q_n is the channel charge per unit area [1b]. Here, M is described above the threshold voltage. With increasing current, M decreases and approaches zero, i.e. temperature and gate voltage increase. θ and η are the mobility modulation factors through γ. The factor θ affects the drain current and degrades mobility due to conventional carriers scattering in the channel and the effect of series resistance. The parameter η is a factor reflecting the drain volage effect, which is reasonable

considering the tiny microscale/nanoscale structures on the edge of the etched mesa. α is the factor lowering the drain current related to the donor concentration, which is around 1.1 in this study. E_F is the quasi Femi level energy of electron, E_C is the conduction bandgap energy, and (E_C-E_D) is the ionization energy for the donor. N_C is the effective density of states in the conduction band and N_d is the donor concentration. g_d is the degeneracy factor of the donor in diamond, which is equal to 2. ε_0 and ε_{dia} are the dielectric constants of vacuum and diamond (5.57), respectively. ε_{ox} is the dielectric constant of Al_2O_3, which is 7 here. n_i is the intrinsic carrier concentration. k_B is the Boltzmann's constant, q is the electron charge, and T is temperature. C_{ox} is the oxide capacitance per unit area. In the present phosphorous doped diamond n-channel MOSFET, C_{ox} is around $1.7x10^{-7}F/cm^2$. The thermal ionization of phosphorous in diamond (E_C-E_D) is set to be 0.57 eV for simulation.

The intrinsic concentration varying with temperature is expressed as

$$n_i = \sqrt{N_C N_V} \exp\left(-\frac{E_g}{2K_B T}\right) \quad \text{(S8)}$$

$$N_C = \frac{2M_C(2\pi m_n k_B T)^{3/2}}{h^3} \quad \text{(S9)}$$

$$N_V = \frac{2M_V(2\pi m_p k_B T)^{3/2}}{h^3} \quad \text{(S10)}$$

Where M_C and M_V are the number of equivalent minima in the conduction band and maxima in the valence band, respectively. For diamond M_C=6 and M_V=1. m_n and m_p are the effective mass around the conduction minimum and the valence band maximum. Here m_n =1.845m_0 and m_p =0.908m_0 . m_0 is the mass of electron. In the saturation region at V_{gs} larger than V_{th} , the gate length is replaced by an effective length. Here, the effective gate length is taken as the same as the device for simplicity.

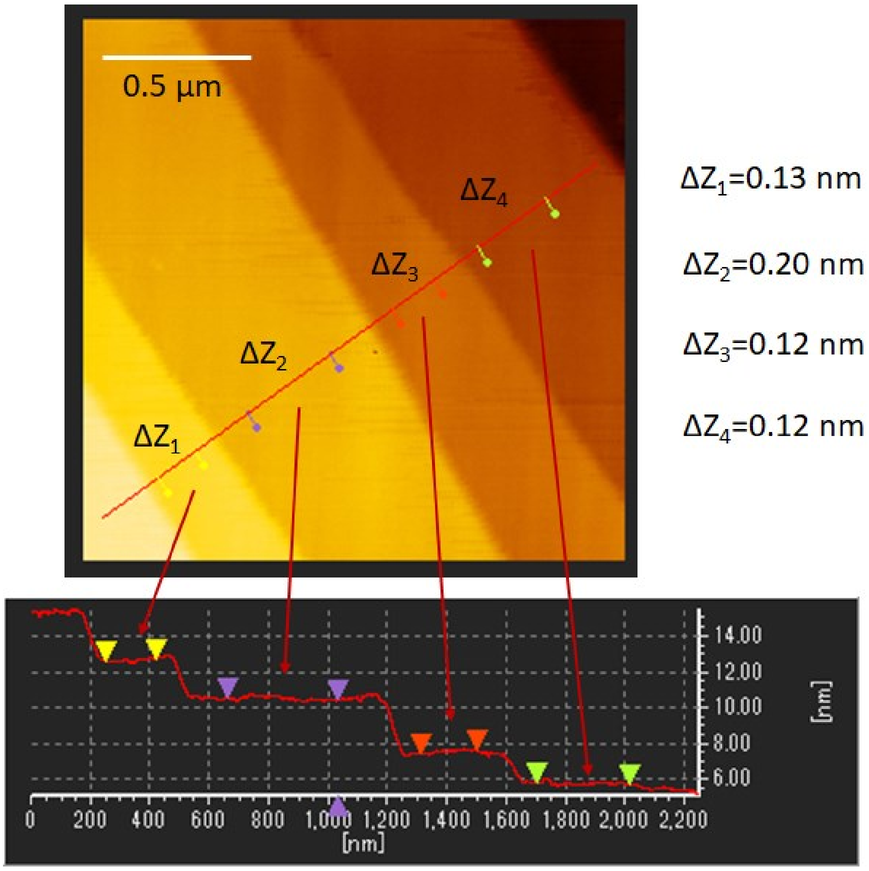

Figure S1. Atomic force microscopy (AFM) image of the n$^-$ diamond epilayer. Terrace structures with atomically smooth surface is observed, indicating the step-flow growth mode. The height difference (ΔZ) is as small as 0.1~0.2 nm, which is due to the off-angle of the miscut substate.

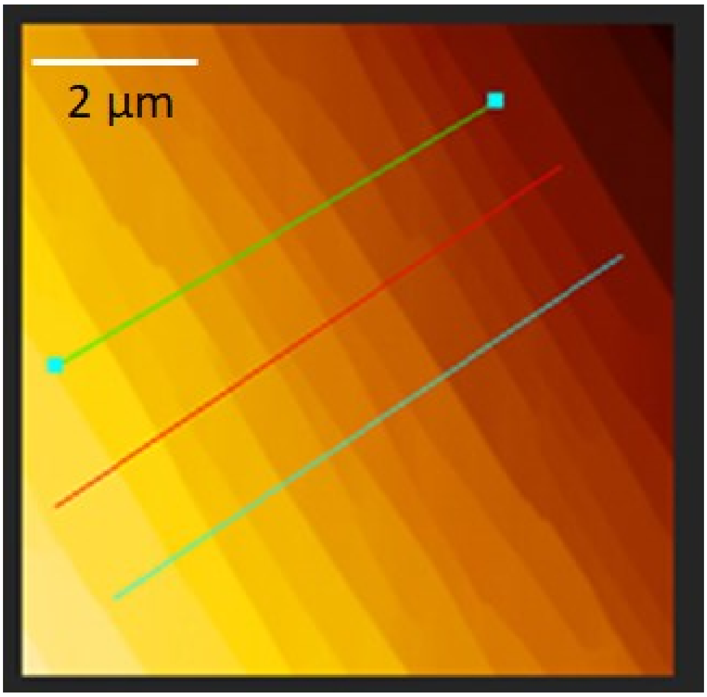

Figure S2. AFM image of the n$^-$ diamond epilayer. The average roughness in the lines region is less than 1 nm despite the steps.

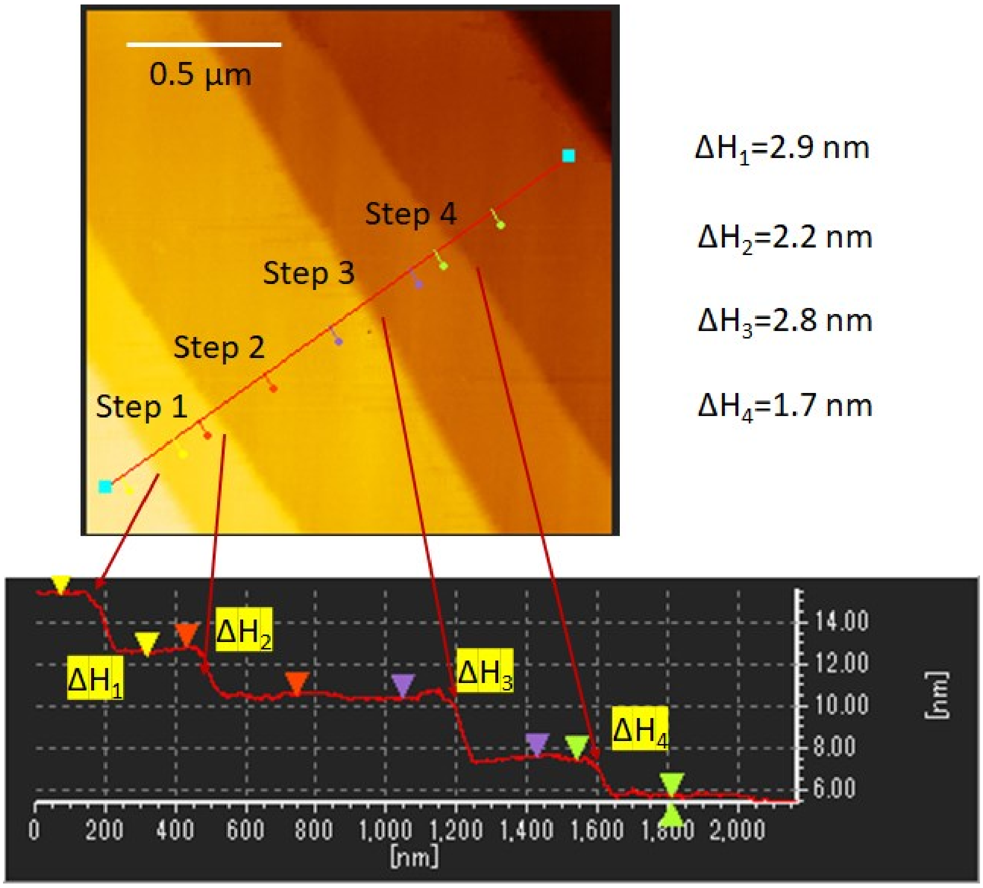

Figure S3. AFM image of the n⁻ diamond epilayer showing the steps. The step height ΔH is within 3 nm.

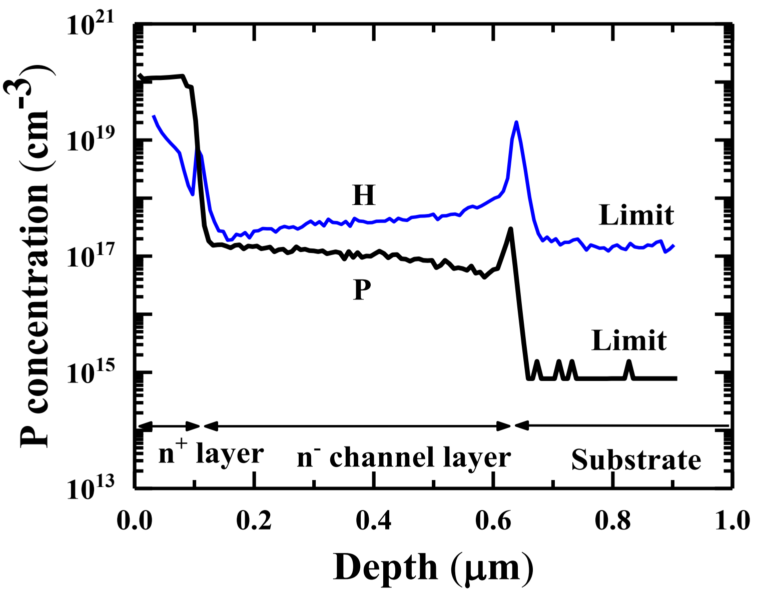

Figure S4. SIMS measurement of the phosphorus and hydrogen concentration on the n^+/n^- diamond layers on the same diamond substrate. The hydrogen content in the n^- channel layer is at the background level.

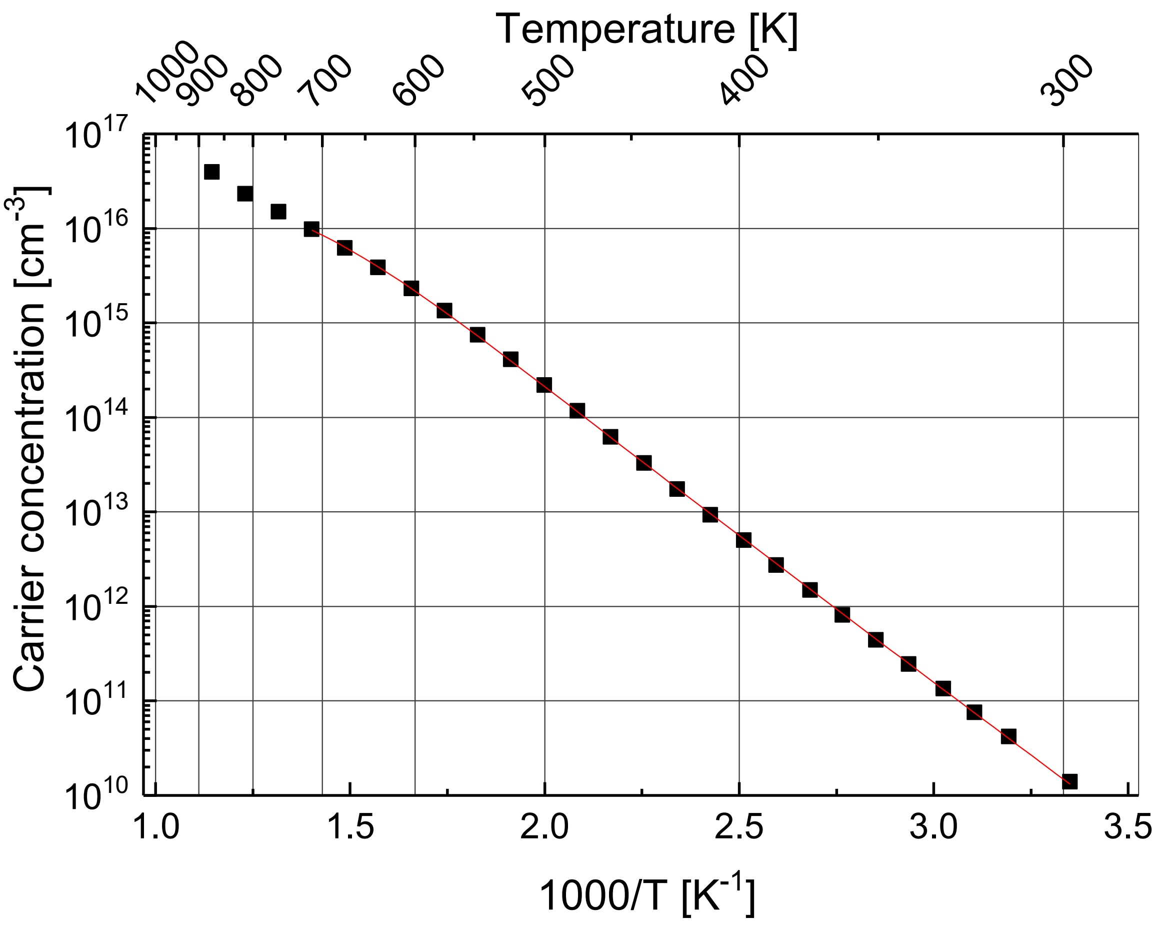

Figure S5. Experimental data of temperature dependent electron density of the n^- diamond epilayer [2].

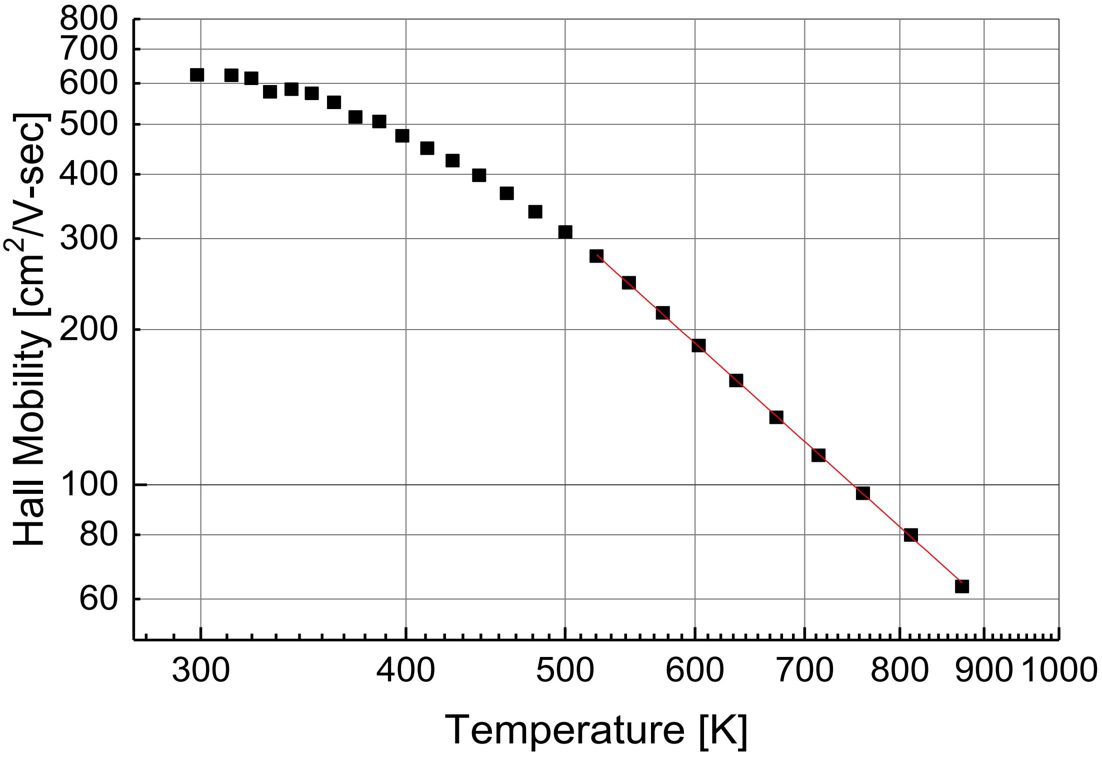

Figure S6. Hall mobility as a variation of the temperature.

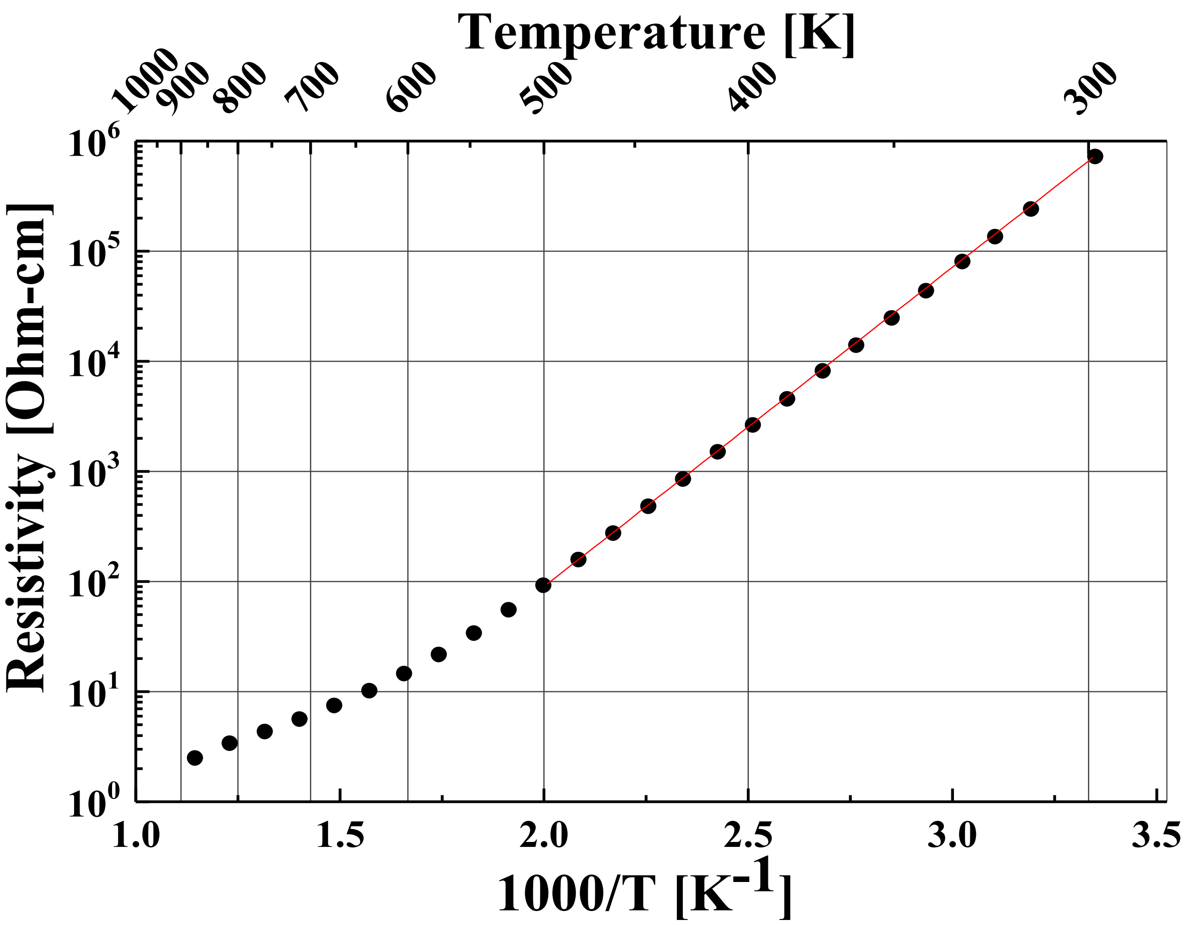

Figure S7. Resistivity as a variation of temperature, showing the activation energy of phosphorous in diamond is around 0.57 eV.

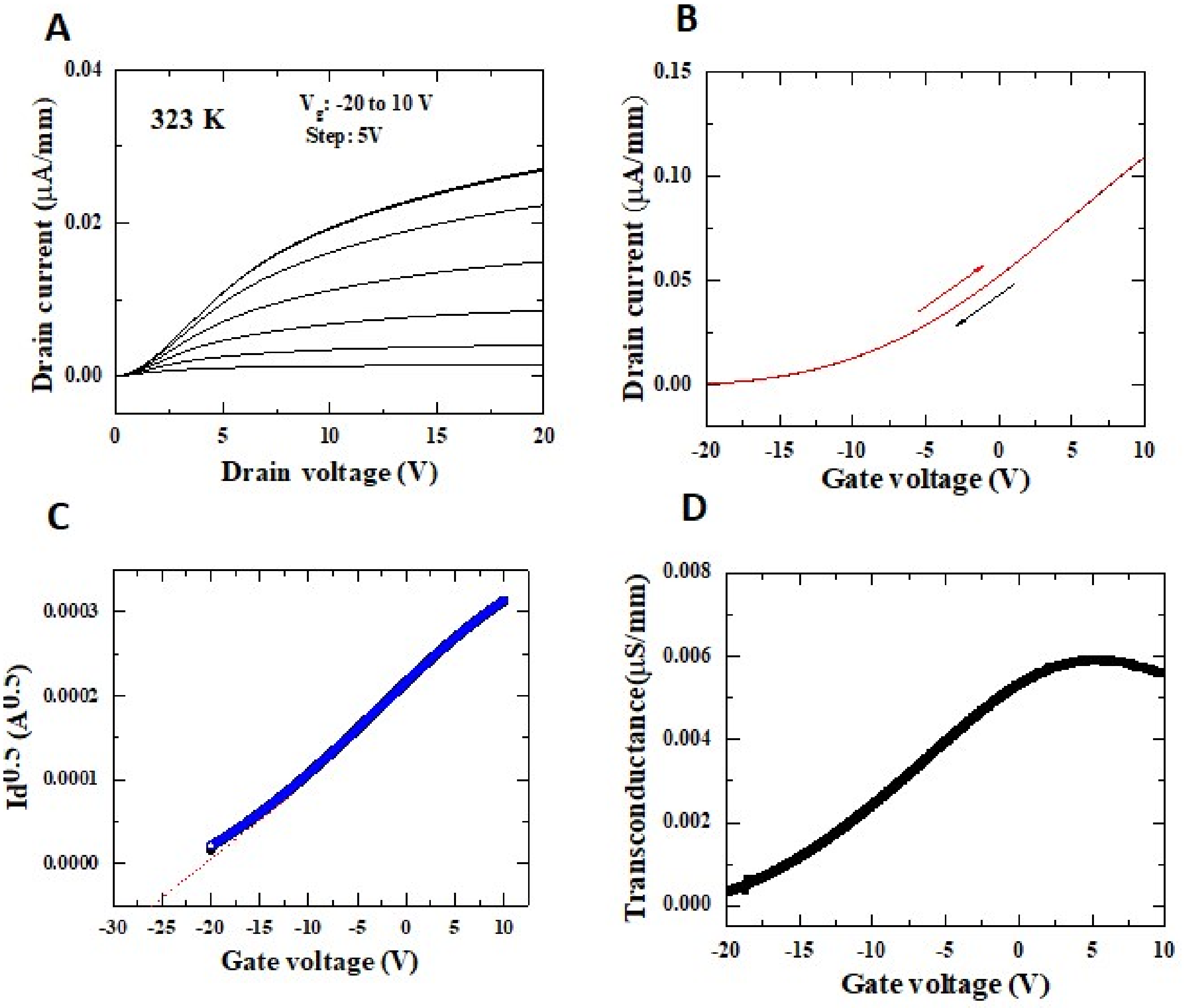

Figure S8. Electrical properties of the n-type diamond MOSFET (device No. 1) presented in the main text at 323 K. (A) Drain current vs drain voltage at different gate voltages. (B) transfer properties. (C) Graphic method for extracting the threshold voltage. (D) Transconductance.

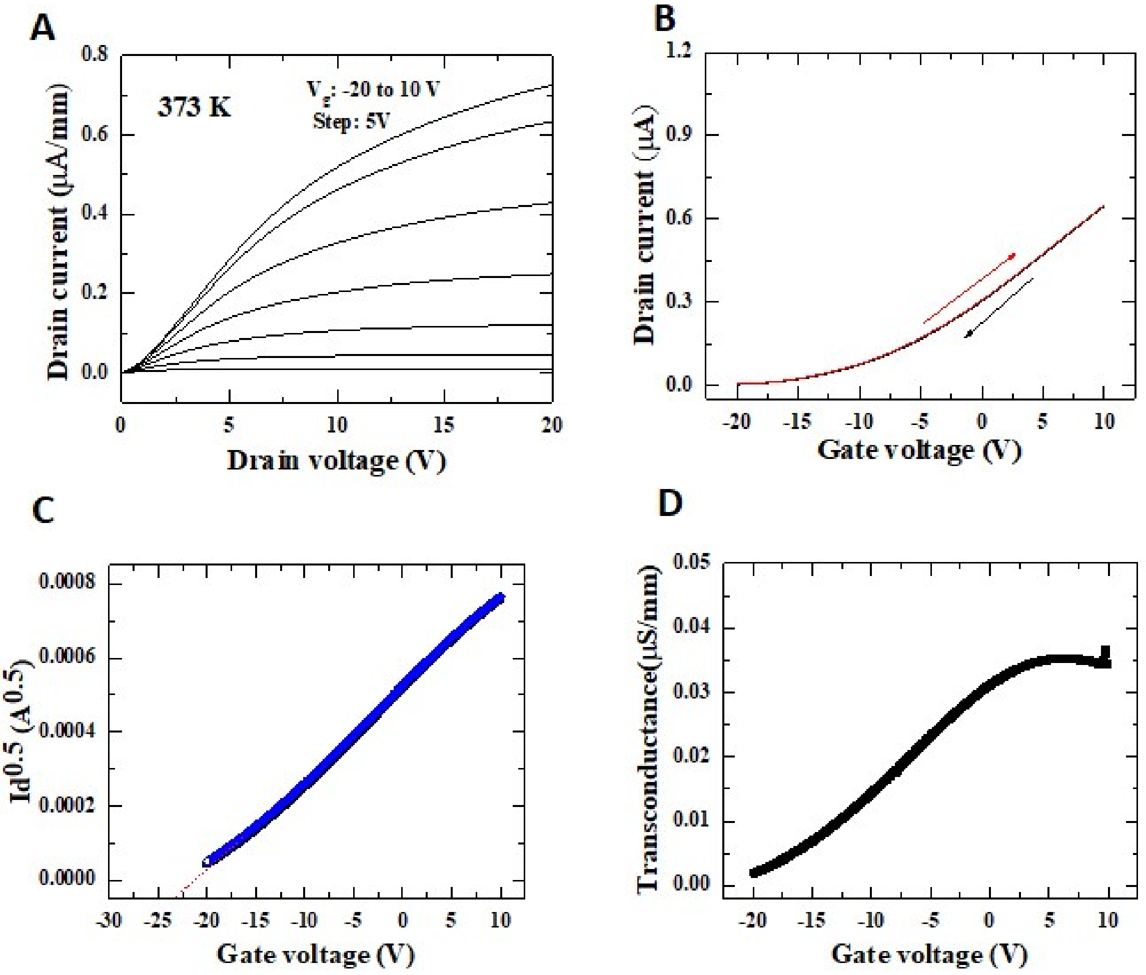

Figure S9. Electrical properties of the n-type diamond MOSFET (device No. 1) presented in the main text at 373 K. (A) Drain current vs drain voltage at different gate voltages. (B) Transfer properties. (C) Graphic method for extracting the threshold voltage. (D) Transconductance.

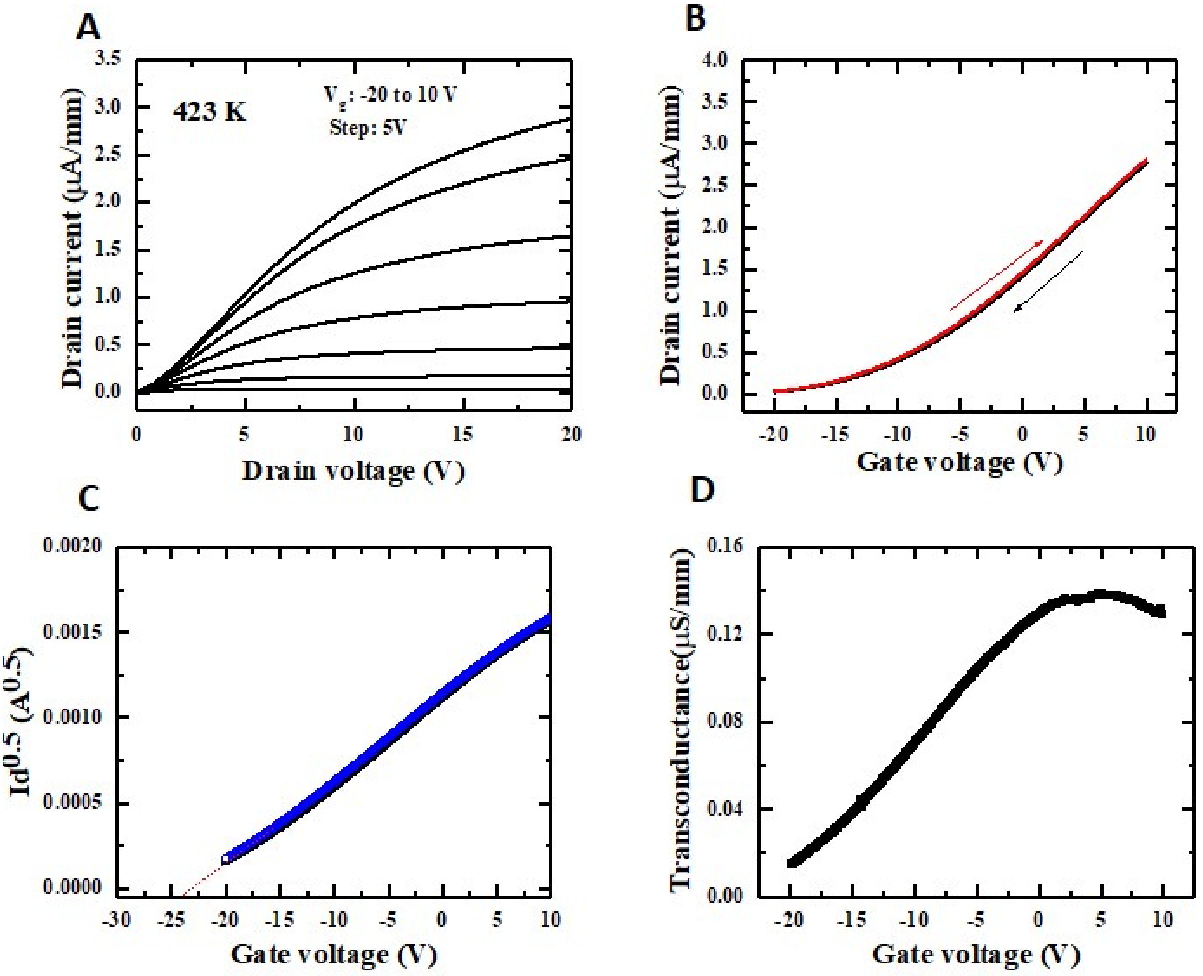

Figure S10. Electrical properties of the n-type diamond MOSFET (device No. 1) presented in the main text at 423 K. (A) Drain current vs drain voltage at different gate voltages. (B) Transfer properties. (C) Graphic method for extracting the threshold voltage. (D) Transconductance.

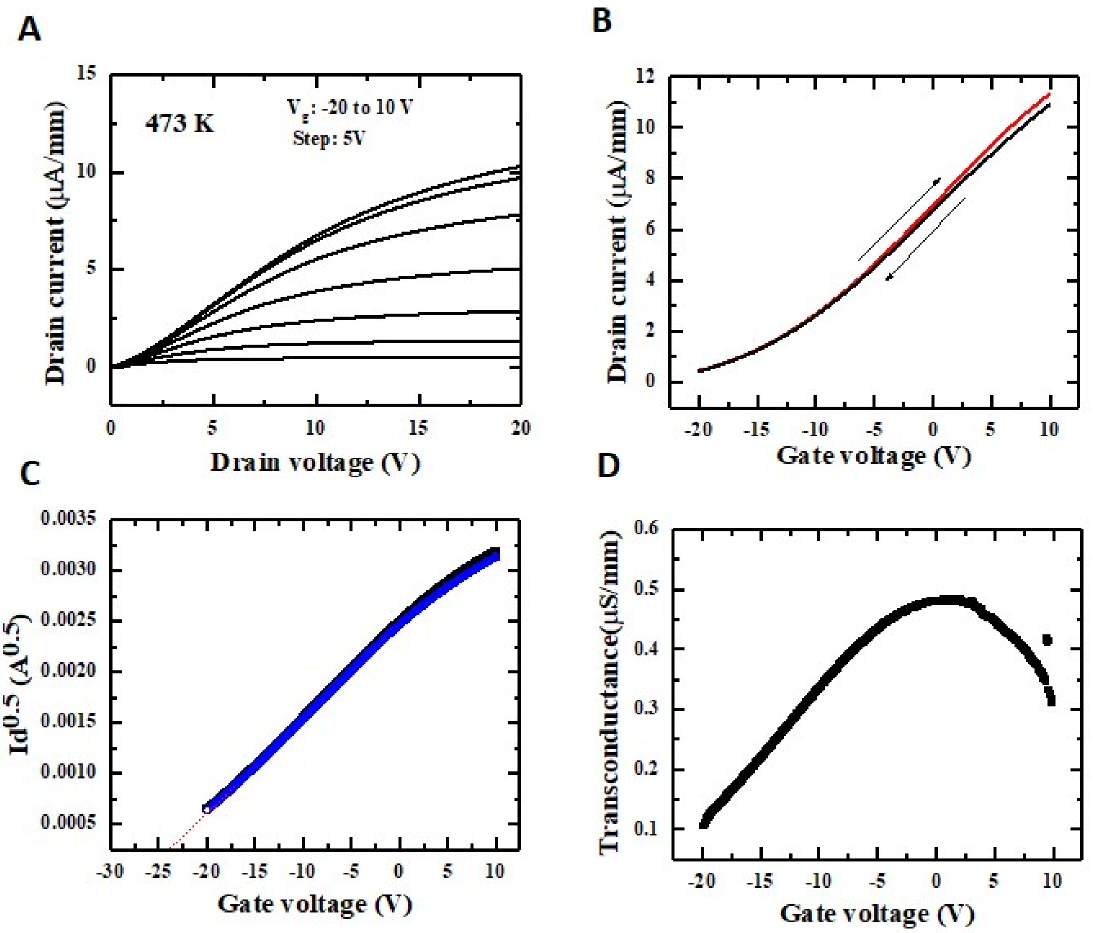

Figure S11. Electrical properties of the n-type diamond MOSFET (device No. 1) presented in the main text at 473 K. (A) Drain current vs drain voltage at different gate voltages. (B) Transfer properties. (C) Graphic method for extracting the threshold voltage. (D) Transconductance.

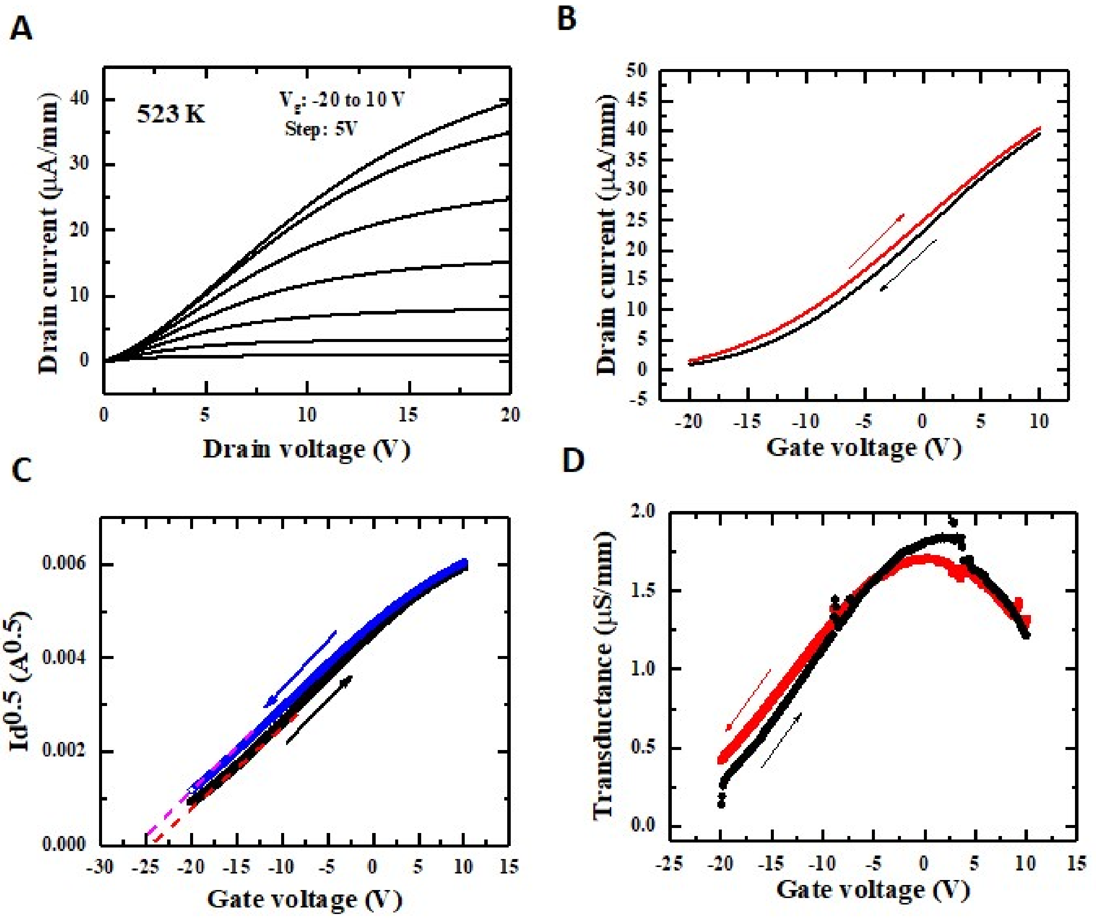

Figure S12. Electrical properties of the n-type diamond MOSFET (device No. 1) presented in the main text at 523 K. (A) Drain current vs drain voltage at different gate voltages. (B) Transfer properties. (C) Graphic method for extracting the threshold voltage. (D) Transconductance.

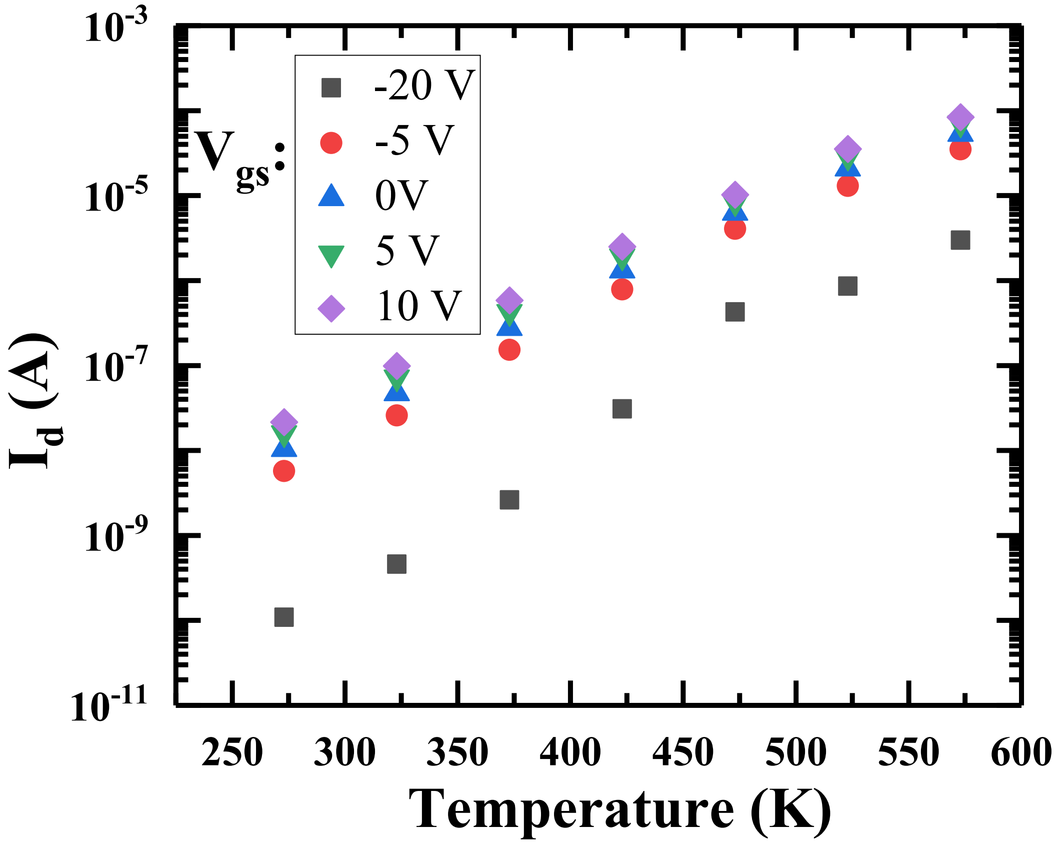

Figure S13. Temperature dependent drain current as temperature for different drain voltages.

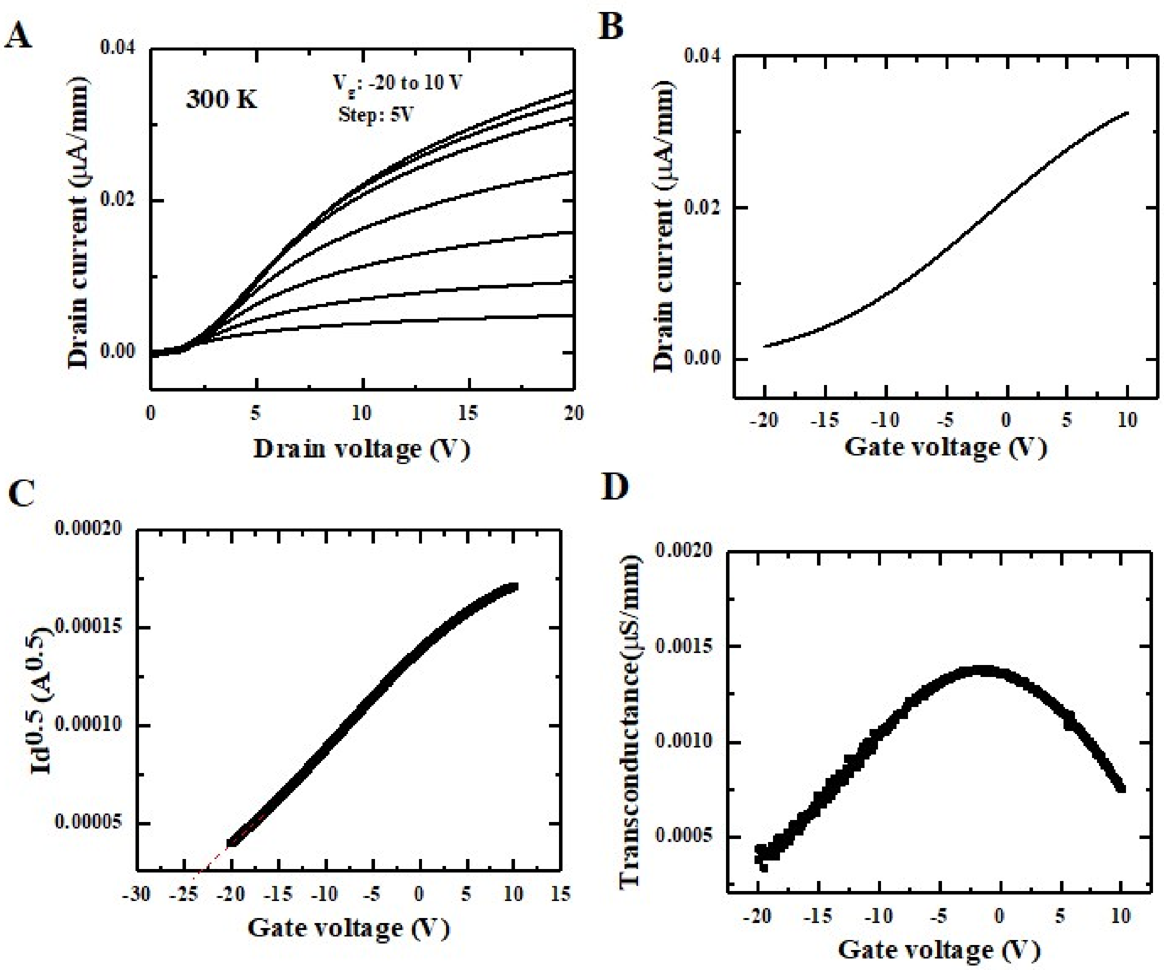

Figure S14. Electrical properties of the n-type diamond MOSFET (device No. 2) with the same dimensions as those of device No.1 at 300 K. (A) Drain current vs drain voltage at different gate voltages. (B) Transfer properties. (C) Graphic method for extracting the threshold voltage. (D) Transconductance.

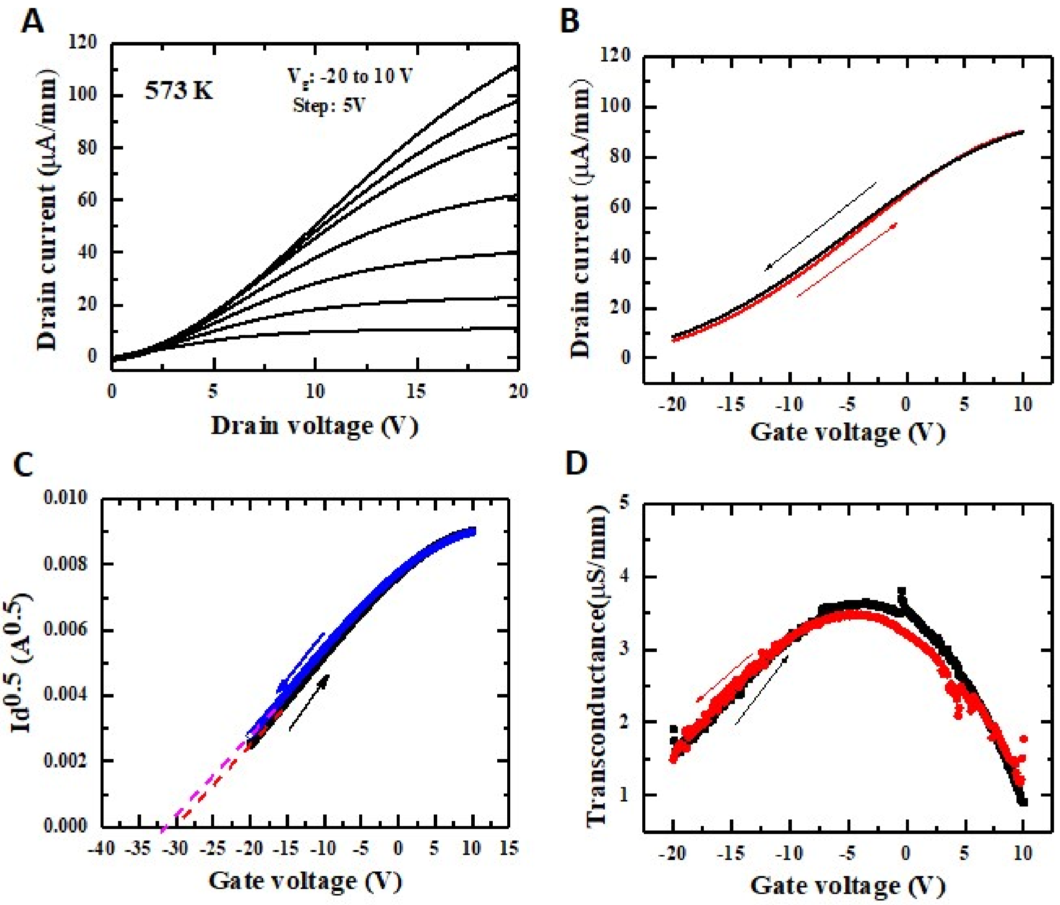

Figure S15. Electrical properties of the n-type diamond MOSFET (device No. 2) with the same dimensions as those of device No.1 at 573 K. (A) Drain current vs drain voltage at different gate voltages. (B) Transfer properties. (C) Graphic method for extracting the threshold voltage. (D) Transconductance.

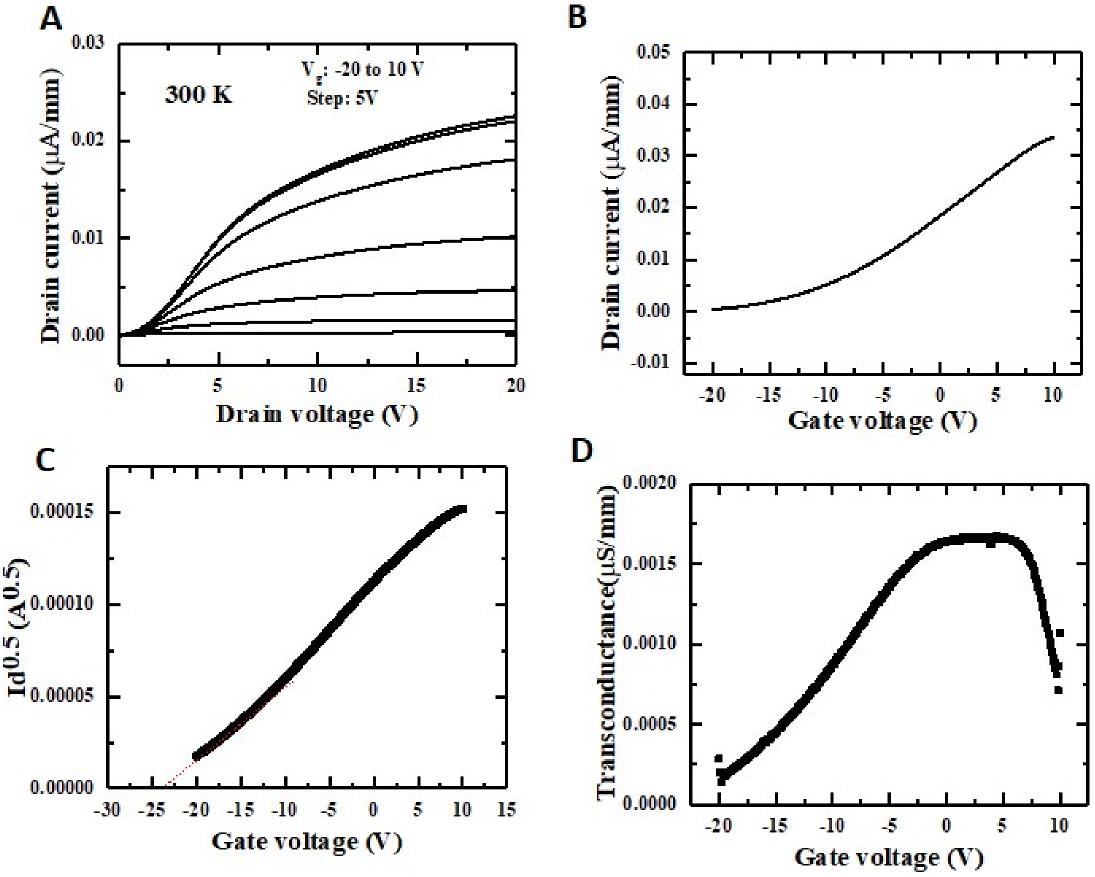

Figure S16. Electrical properties of the n-type diamond MOSFET (device No. 4) with the circular geometry with a gate length 10 μm, source-gate and drain-gate distance of 10 μm at 300 K. (A) Drain current vs drain voltage at different gate voltages. (B) Transfer properties. (C) Graphic method for extracting the threshold voltage. (D) Transconductance.

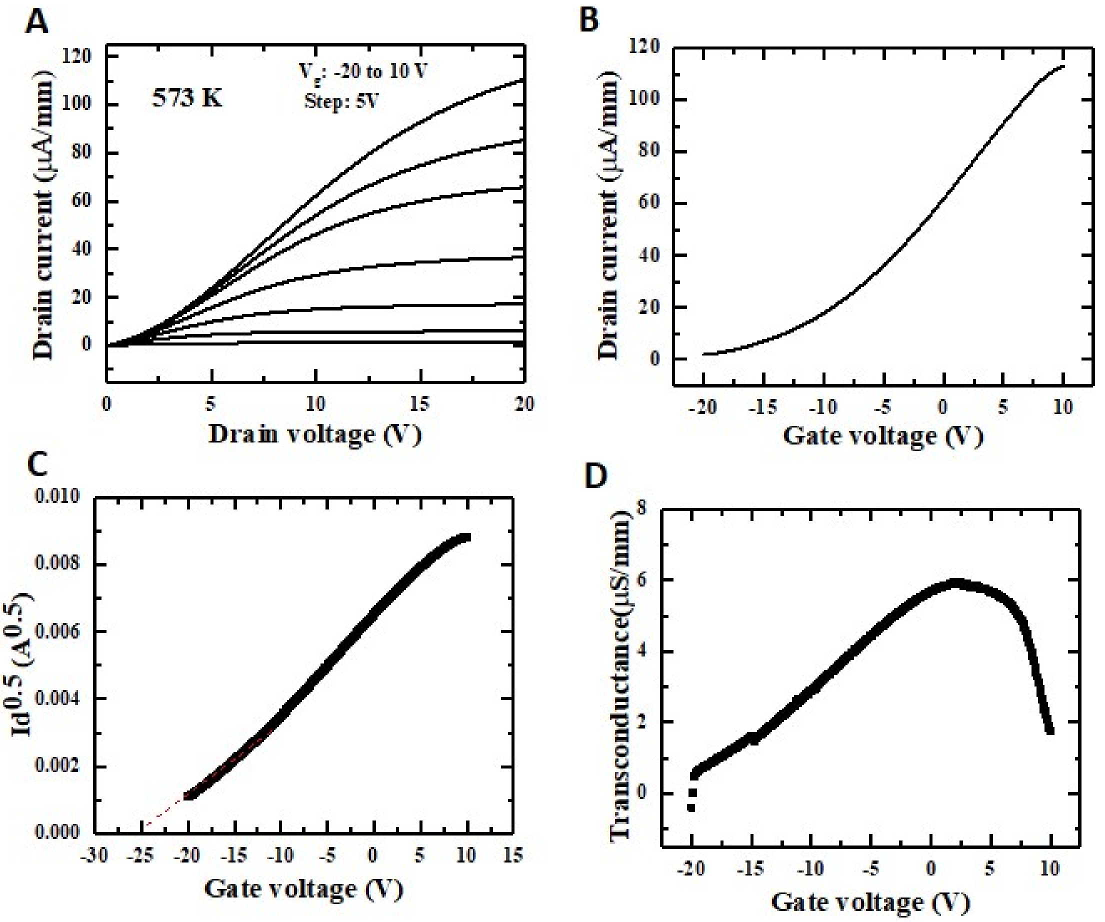

Figure S17. Electrical properties of the n-type diamond MOSFET (device No. 4) with the circular geometry with a gate length 10 µm, source-gate and drain-gate distance of 10 µm at 573 K. (A) Drain current vs drain voltage at different gate voltages. (B) transfer properties. (C) graphic method for extracting the threshold voltage. (D) Transconductance.

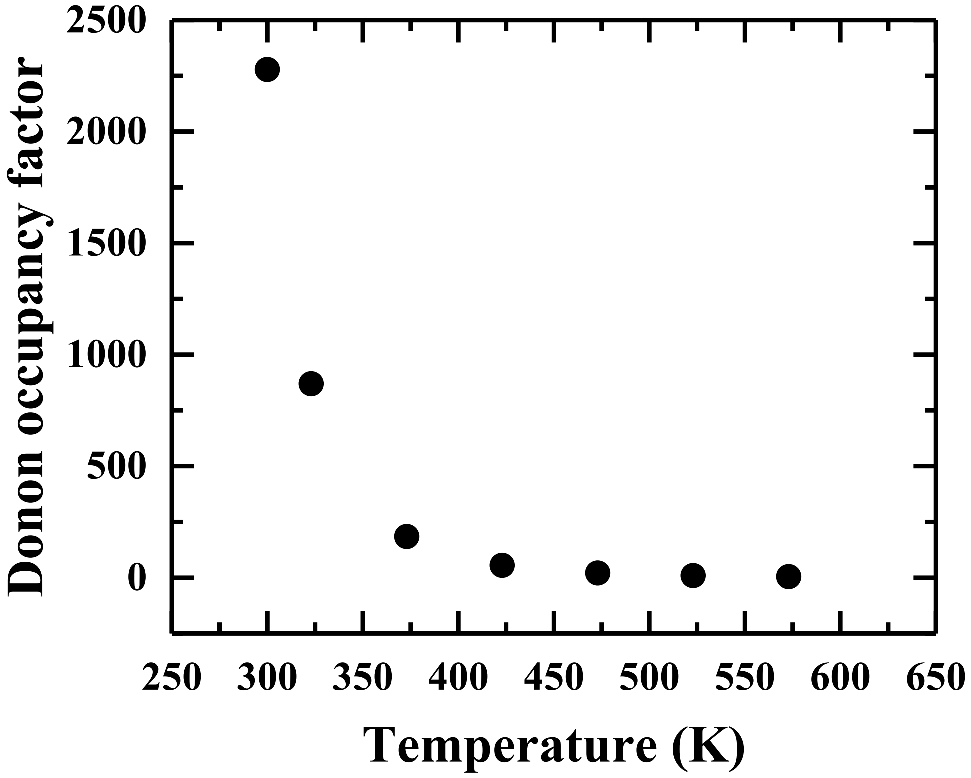

Figure S18. The donor occupancy factor M at different temperatures. M decrease from 2278 at 300 K to around 4 at 573 K.

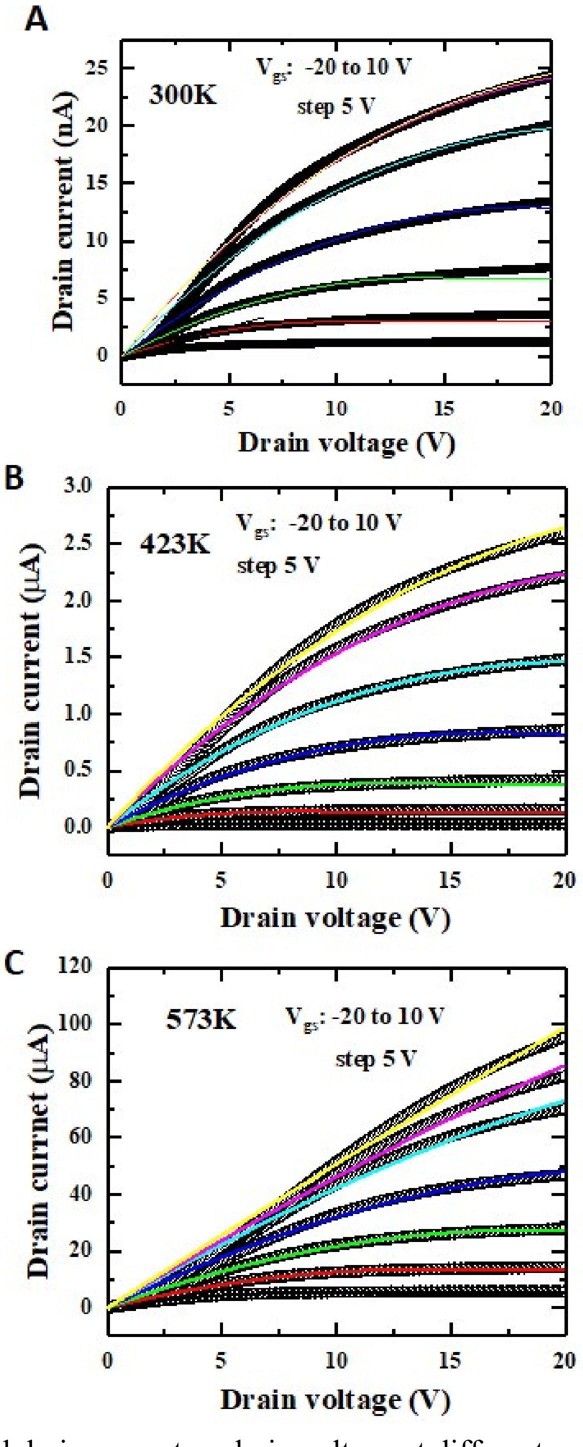

Figure S19. Simulated drain current vs drain voltage at different gate voltages and different temperatures. (A) 300K, (B) 423 K, (c) 573 K.

Table S1.

Drain currents of the n-type MOSFETs studied in this work. The $I_{d,sat}$ is for the drain current at V_{ds}=20V and V_{gs}=10V. The source-gate spacing L_{sg} is equal to gate-drain spacing L_{dg}. Devices No.1 to No.3 are rectangular type and device No.4 is circular type.

Device	L_g (μm)	W_g (μm)	L_{sg} (μm)	$I_{d,sat}$@300K(μA/mm)	$I_{d,sat}$@573K (μA/mm)
No.1	5	900	10	0.027	105
No.2	5	900	10	0.035	112
No.3	10	900	10	0.02	80
No.4	5	691	10	0.022	110

References

[1] a) H. I. Hanafi, L. H. Camnitz, A. J. Dally, *IEEE Journal of Solid-State Circuits* **1982**, *17* (5), 882, https://doi.org/10.1109/JSSC.1982.1051835; b) K. A. Wilson, P. L. Tuxbury, R. L. Anderson, *IEEE Transactions on Electron Devices* **1986**, *33* (11), 1731, https://doi.org/10.1109/T-ED.1986.22735.

[2] S. Koizumi, M. Suzuki, *physica status solidi (a)* **2006**, *203* (13), 3358, https://doi.org/https://doi.org/10.1002/pssa.200671407.
